# S5. Proffered paper: Maintenance therapy of metastatic colorectal carcinoma with the TLR-9 agonist MGN1703: clinical and immunological predictive pretreatment factors of activity in the IMPACT trial

**DOI:** 10.1186/2051-1426-2-S2-I2

**Published:** 2014-03-12

**Authors:** H Schmoll, BW Wittig, DA Arnold, JRK Riera-Knorrenschild, DN Nietsche, HK Kroening, FM Mayer, JA Andel, RZ Ziebermayr, WS Scheithauer

**Affiliations:** 1 Universitätsklinikum HalleOnkologie/Hä, Halle, Germany; 2 Freie Universität Berlin, Foundation Institute Molecular Bioogy and Bioinformatics, Berlin, Germany; 3 Tumor Biology Center, Medical Oncology, Freiburg, Germany; 4 University Clinic Giessen/Marburg, Haematology and Oncology, Marburg, Germany; 5 Hospital Barmherzige Schwestern, Internal Medicine, Linz, Austria; 6 Schwerpunktpraxis, Hematology and Oncology, Magdeburg, Germany; 7 University Tübingen Medical Center, Internal Medicine II, Tübingen, Germany; 8 Landeskrankenhaus Steyr, Internal Medicine II, Steyr, Austria; 9 Elisabethinen, Hematology with stem cell transplantation and medical oncology, Linz, Austria; 10 Medical University Vienna, Internal medicine and comprehensive cancer center, Vienna, Austria

## Background

MGN1703 is a synthetic DNA-based immunomodulator acting as TLR-9 agonist which has shown preclinical activity in metastatic colorectal carcinoma (mCRC) as well as a good safety profile in patients with metastatic solid tumours in a Phase 1 trial. The IMPACT trial was conducted to assess clinical efficacy, safety, and immunological effects of MGN1703 as maintenance therapy twice weekly s.c. vs. placebo.

## Methods

IMPACT was an international randomised (2:1) double-blind placebo-controlled phase 2 trial in patients with mCRC who achieved disease control (CR, PR, SD) after 4.5 to 6 months of 1st-line induction chemotherapy with FOLFOX/XELOX or FOLFIRI +/- bevacizumab. Due to slow recruitment the trial was prematurely closed in May 2012 after randomisation of 59 out of 129 planned patients (43 received MGN1703, 16 placebo).

## Results

There was evidence of a superior effect of MGN1703 compared with placebo. The hazard ratio (HR) for the primary endpoint PFS on maintenance treatment group was 0.55, (p=0.040) on local assessment and 0.56 (p=0.070) by independent radiological review. Notably, at time of study closure 4 patients receiving MGN1703 were still free of progression and continued treatment in compassionate use protocols.

Exploratory uni- and multivariate Cox regression analyses showed a possibly predictive effect of baseline CEA level and tumour size change during first-line induction therapy. HR was 0.07 (p<0.0001) for patients with normal CEA level and 0.39 (p=0.005) for patients with an objective response to induction chemotherapy.

A predefined analysis was performed on immunological cell populations at baseline and during the study. This allowed to confirm activation of innate immune system effector cells in patients receiving MGN1703. Cox regression and receiver operating characteristic (ROC) analyses identified the presence at baseline of activated NKT-cells (CD3+, CD56+, CD69+) as potentially predictive of benefit from MGN1703 treatment. The HR was 0.27 (p=0.007) using a cut-off value for activated NKT-cells of 3.08%.

## Conclusions

After induction chemotherapy for mCRC, maintenance with MGN1703 is associated with improved PFS compared to placebo and low toxicity.

We found preliminary evidence that pretreatment CEA plasma levels, tumour response and activated NKT cells counts may allow identifying patients benefiting most from MGN1703 maintenance therapy. A confirmatory clinical study in patients with mCRC is planned to start in 2014.

